# Formulation Development and Evaluation of Indian Propolis Hydrogel for Wound Healing

**DOI:** 10.3390/gels9050375

**Published:** 2023-05-01

**Authors:** Harshad S. Kapare, Prabhanjan S. Giram, Sadhana S. Raut, Hemant K. Gaikwad, Ana Cláudia Paiva-Santos

**Affiliations:** 1 Dr. D. Y. Patil Institute of Pharmaceutical Sciences and Research, Pimpri, Pune 411018, India; 2 Department of Pharmaceutical Sciences, The State University of New York, Buffalo, NY 14214, USA; 3 STES Sinhgad College of Pharmacy, Vadgaon (BK), Pune 411041, India; 4 Department of Pharmaceutical Technology, Faculty of Pharmacy of the University of Coimbra, University of Coimbra, 3004-531 Coimbra, Portugal; 5 REQUIMTE/LAQV, Group of Pharmaceutical Technology, Faculty of Pharmacy of the University of Coimbra, University of Coimbra, 3004-531 Coimbra, Portugal

**Keywords:** burn wound, excision wound, hydrogel, incision wound, Indian propolis, natural product, wound healing

## Abstract

Flavonoids and polyphenolic compounds play a key role in wound healing cycle modulation. Propolis, a natural bee product, has been widely reported as an enriched source of polyphenols and flavonoids as important chemical constituents and for its wound healing potential. The goal of this study was to develop and characterize a propolis-based polyvinyl alcohol (PVA) hydrogel composition with wound healing potential. To understand the impacts of critical material attributes and process parameters, formulation development was carried out using a design of experiment approach. A preliminary phytochemical analysis of Indian propolis extract showed the presence of flavonoids (23.61 ± 0.0452 mg equivalent of quercetin/g) and polyphenols (34.82 ± 0.0785 mg equivalent of gallic acid/g), both of which aid in wound healing and skin tissue regeneration. The pH, viscosity, and in vitro release of the hydrogel formulation were also studied. The burn wound healing model results revealed significant (*p* < 0.0001) wound contraction by propolis hydrogel (93.58 + 0.15%) with rapid re-epithelialization relative to 5% *w*/*w* povidone iodine ointment USP (Cipladine^®^) (95.39 + 0.16%). The excision wound healing model confirms significant (*p* < 0.0001) wound contraction by propolis hydrogel (91.45 + 0.29%) with accelerated re-epithelialization comparable to 5% *w*/*w* povidone iodine ointment USP (Cipladine^®^) (94.38 + 0.21%). The developed formulation offers promise for wound healing, which may be investigated further for clinical research.

## 1. Introduction

Propolis (bee glue) is a resinous and sticky material that bees acquire to build and alter their hives [1]. Propolis has almost 300 chemical constituents, including polyphenols, amino acids, sesquiterpenes, quinine, coumarins, steroids, and some inorganic substances. Unlike many natural remedies, propolis has a comprehensive database on its biological activity and toxicity, indicating that it has a wide range of pharmacological activities such as antibacterial, antifungal, antiviral, and anticancer activities in many traditional texts such as ayurveda, sidhha, Chinese medicine, and so on [2]. To extract the physiologically active components of propolis, several extraction procedures were applied. Extraction procedures such as conventional maceration, ultrasonic, and microwave-aided extraction were used to enrich the active components and hence increase pharmacological response [3,4,5,6].

Wound healing is a multistage tissue regeneration process that consists of inflammatory, proliferative, and remodeling stages [7]. To enhance rapid tissue regeneration, dead tissue is replaced with fresh healthy cells, which reduces the acute inflammatory reactions induced by damage and necrosis to the surrounding matrix. Bacterial infections (*S. auresus*) at the injury site cause a delay in the wound healing process. Traditional medical systems such as Ayurveda, siddha, and Chinese medicine have emphasized natural products in the treatment of injury [8,9]. An increase in the frequency of physical injuries and accidental cases necessitates the use of wound care products. A survey analysis found roughly 8.2 million people suffering from wounds with or without infections, with medication costs ranging from USD 28.1 billion to USD 96.8 billion worldwide. The wound care products industry is anticipated to reach USD 15–22 billion by 2024 [10].

Despite the well-established wound healing potential of propolis [11,12], its therapeutic usefulness has received little attention owing to a variety of limitations [13,14,15,16]. Modern formulation methods for the administration of synthetic or natural materials in wound healing treatment have evolved. A hydrogel is a three-dimensional hydrophilic polymeric network that can absorb large amounts of water or biological fluid [17]. Because of physical and chemical cross-linking, this network structure is insoluble in nature, providing physical integrity. Because of their thermodynamic compatibility, these hydrogel structures swell in an aqueous environment. In terms of water content, softness, and other properties, these hydrogels are structurally and functionally similar to real tissues. As a result, hydrogels have shown their usefulness as contact lenses, artificial skin materials, and for medication delivery [18,19,20]. PVA is well proven as a wound dressing material to produce the required moist wound environment, enhance the dressing’s physical qualities, and speed up wound healing. Moreover, PVA can be utilized for medicine administration and wound healing and can be blended with various organic and inorganic components [21]. Because of their stability, biocompatibility, water holding capacity, lack of toxicity, and resistance to biological aging, PVA-based hydrogel systems have shown good wound healing potential [22]. Despite some studies reporting propolis formulations for wound healing potential, detailed investigations for formulation parameters and wound healing activities on burn, incision, and excision wounds need to be evaluated [23]. The goal of this study would be to develop a PVA-based hydrogel formulation of Indian propolis for wound healing potential, which will serve as a drug delivery system with a combination of synergistic activities of propolis constituents and the benefits of PVA hydrogel for improved formulation characteristics and therapeutic performance.

## 2. Results and Discussion

### 2.1. Extraction and Standardization of Propolis

The propolis extract was standardized in terms of specific wound healing indicators using a developed and validated RP HPLC method [24]. Total polyphenols, total flavonoids, and pesticidal content were also evaluated in the extract. Total polyphenol content was 34.82 + 0.072 mg equivalent of gallic acid/g and 23.61 + 0.045 mg equivalent of quercetin/g, respectively. A total of 113 pesticides were confirmed to be absent or within limits in the sample [24].

### 2.2. Formulation Optimization

From the initial screening for polyvinyl alcohol at different concentration ranges from 5 to 15%,the concentrations from 8% to 10% were observed with desirable ranges of responses in terms of viscosity (29,000–32,000 cps) as well as in vitro drug release (93.25–96.79%), so these concentrations were selected for the further experimental design. The incorporation of Indian propolis extract into the prepared gel bases resulted in a brownish hue with a pH of 5.4, which is within the acceptable pH range (5–5.5) for topical treatments. Table 1 and Table 2 shows details of the 3^2^ factorial design that was used to optimize the formulation.

#### Effect on Drug Release and Viscosity

Polymer concentration and stirring speed were identified as critical parameters needing optimization in the formulation development process to obtain a final optimized formulation with desirable characteristics.

The responses of these batches are shown in Table 2. Multiple regression analysis was conducted using Design Expert^®^ Version 10.0.

Positive coefficients of the main terms X1 and X2 for in vitro drug release and negative coefficients of the main term X2 for viscosity indicated a favorable effect.

**Figure 1:**
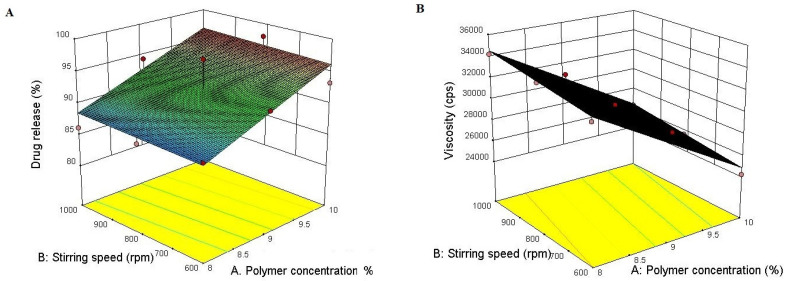
Showing 3D response surface plots for in vitro drug release (**A**) andviscosity (**B**) showing the influence of the concetration of polymer and stirring speed.

The polymer concentration and stirring speed both had a linear effect on in vitro drug release and viscosity as seen in the response surface plot (Figure 1).

**Figure 2:**
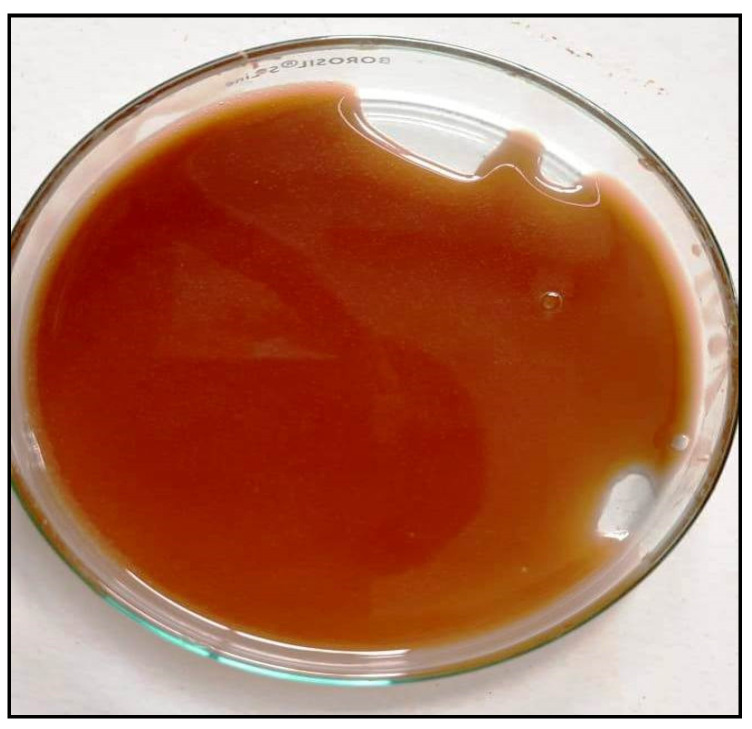
Propolis Hydrogel.

Figure 1 also reveals that both polymer concentration and stirring speed show desirable effects with their optimized combination as shown in the solution for optimum batch selection, where the highest desirability of 1.000 was obtained at 9.564% polymer concentration and 739.21 rpm for hydrogel formulation at the desired in vitro drug release and viscosity, so F2 was selected as an optimized formulation and further evaluated for various parameters. Figure 2 shows the image of the representative optimized batch of propolis hydrogel.

### 2.3. Visual Appearance

The prepared formulation was inspected visually and the results of same were observed as given below in Table 3.

The viscosity of developed formulation batches was analyzed and observed in the range of 24,200 to 34,200 cps. Viscosity was evaluated as a dependent variable to optimize the formulation batch. The pH of the gel formulation was determined using a digital pH meter and it was found to be 5.4 ± 0.246 pH; a formulation in the slight acidic range favors intact drug delivery through the skin as it remains acidic at the surface as well as in different skin layers.

### 2.4. FT-IR Study

The FTIR spectrum of propolis extract was recorded in the range of 400–4000 cm^−1^. Various modes of vibrations were identified and assigned to determine the different functional groups present in the propolis extract.

**Figure 3:**
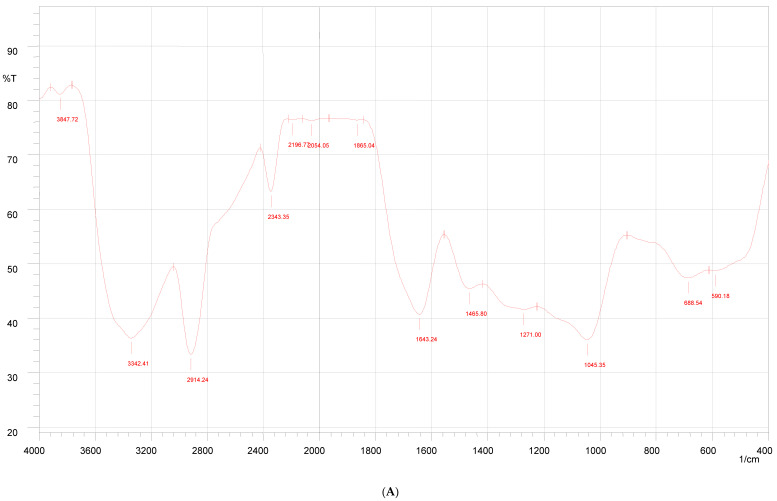

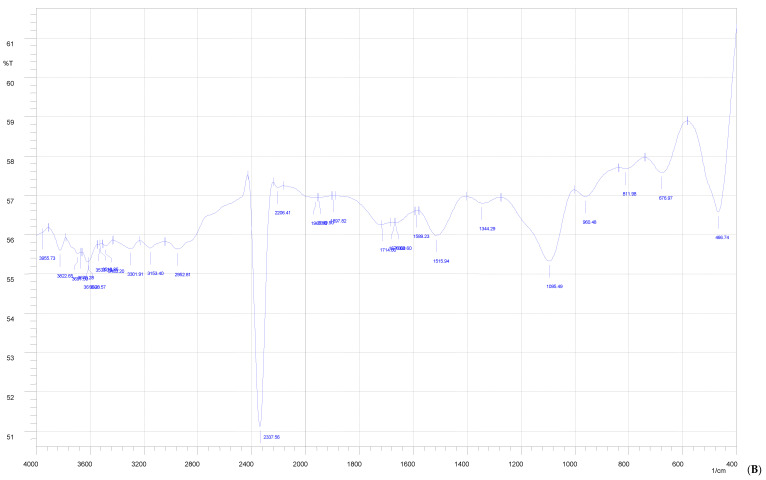

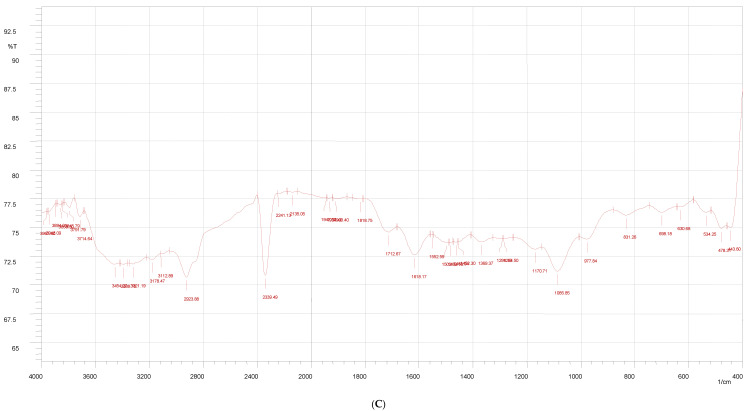
FTIR of Indian propolis (**A**), PVA (**B**), and propolis hydrogel formulation (**C**).

As shown in Figure 3, the FT-IR spectrum of propolis extract showed various peaks at 3571.52 cm^−1^, 3541.63 cm^−1^, 3485.7 cm^−1^, and 3333 cm^−1^ showing –OH stretching. The band obtained at 3083.62 cm^−1^ was assigned to C–H stretching. The strong and narrow peaks at 1639.2 cm^−1^, 1594.8 cm^−1^, and 1164.7 cm^−1^ were attributed to C=O, C=C, and C–O stretching, respectively.

The FTIR spectrum of the propolis hydrogel formulation shows peaks of –OH stretching at 3571.52 cm^−1^ and 3541.63 cm^−1^; C–H stretching at 3083.62 cm^−1^, 1639.2 cm^−1^, 1594.8 cm^−1^, and 1164.7 cm^−1^ with decreased intensity; and prominent peaks at 3325 cm^−1^ due to the O–H stretching vibration, 2937 cm^−1^ due to the C–H stretching vibration, 1419 cm^−1^ due to C–O carbonyl stretching, and 1245 cm^−1^ due to the C–H bending vibration of CH 2, confirming the presence and distribution of propolis in the hydrogel formulation.

### 2.5. DSC

**Figure 4:**
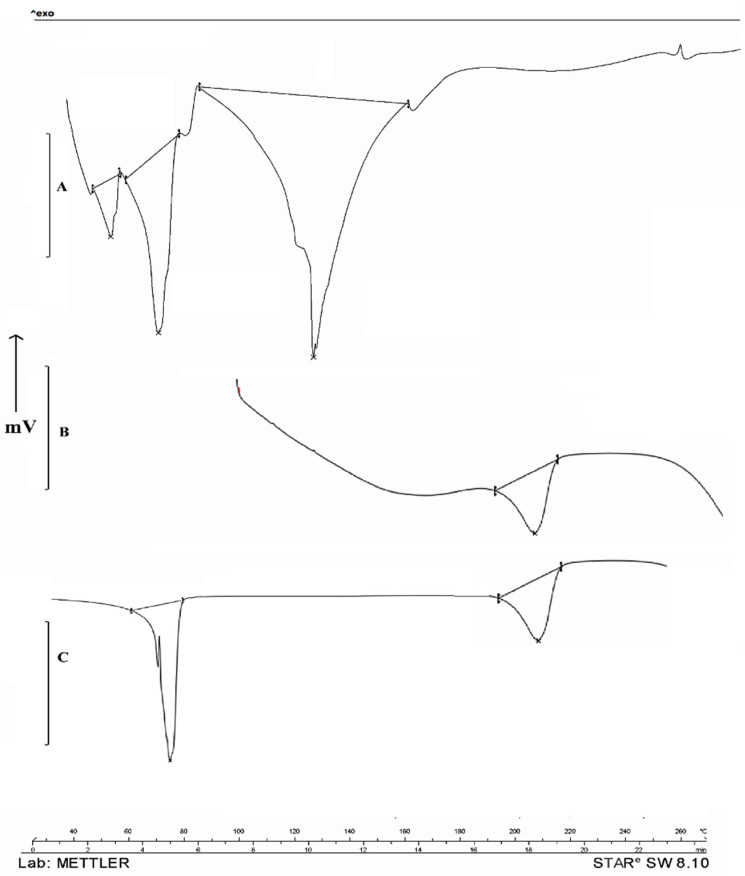
DSC Study of Indian propolis showed melting temperatures (T_m_) at 40.11 °C, 56.81 °C, and 112.43 °C (**A**). PVA showed its typical melting temperature (T_m_) at 221.61 °C (**B**) and propolis hydrogel formulation showed absence of melting peaks at 40.11 °C and 56.81 °C and reduced intensity at 112.43 °C (**C**).

DSC studies were performed to study the nature of propolis in a hydrogel formulation (Figure 4). Propolis with the presence of polyphenols and flavonoids showed sharp melting transitions at 40.11 °C, 56.81 °C, and 112.43 °C. PVA showed its typical melting temperature (T_m_) of 221.61 °C. Propolis hydrogel showed an absence of peaks at 40.11 °C and 56.81 °C, and the reduced intensity at the 112.43 °C melting peak indicates molecular dispersion in the hydrogel formulation.

### 2.6. In Vitro Drug Release Study

**Figure 5:**
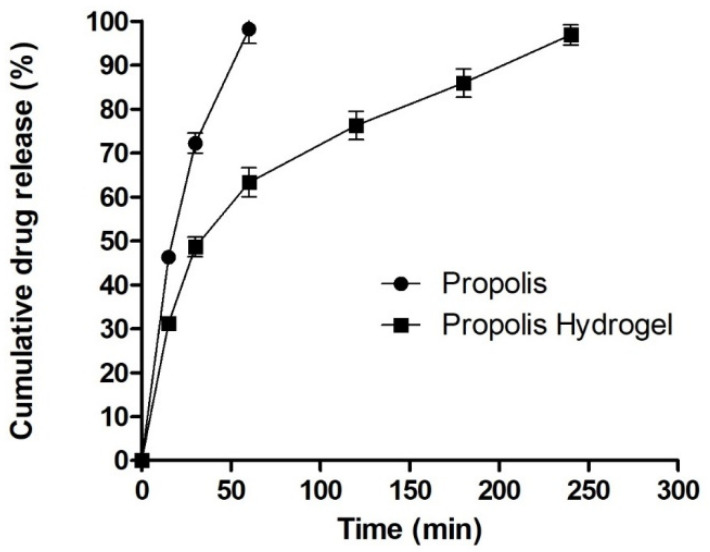
In vitro drug release study.

The drug release pattern from the hydrogel formulation is closely related to the drug interaction and matrix structure. The propolis release pattern obtained from the hydrogel formulation is presented in Figure 5. The release study reveals a biphasic drug release pattern where an initial burst release pattern of the drug was observed during first 40 min, with about 40% of the drug being released, followed by a comparatively slow release up to 4 h. The pattern might be due to the dense hydrogel matrix with strong physical drug–hydrogel interactions which reduces drug release through hydrogel as compared with pure drug release, which releases in 70 min with a 98.22% release. The detailed mechanism with effects of pore size and water absorption capacity can be further investigated.

### 2.7. Acute Dermal Toxicity Study

Study observations showed no death or clinical alterations in terms of various parameters observed as mentioned in the methods section in terms of skin, respiratory, circulatory, and autonomic and central nervous systems. Hair shedding, tremors, seizures, salivation, sedation, and drowsiness were not observed. Study results reveal the extract was found to be not toxic at a dose of 2000 mg/kg. The significant difference (*p*  <  0.05) in percent body weight (weight gain) observed upto day 14 suggests no systemic toxic effects or any organic damages.

### 2.8. Wound Healing Studies of Hydrogel Formulation

#### 2.8.1. Burn Wound Model

**Figure 6:**
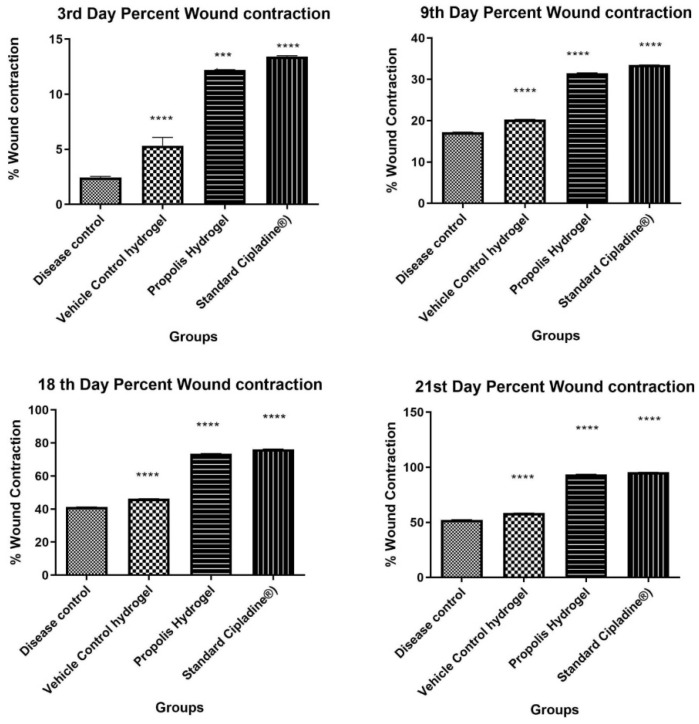
Percent wound contraction at time intervals of the 3rd, 9th, 18th, and 21st days (Data were analyzed by one-way ANOVA followed by Tukey–Kramer Multiple Comparison Test *** *p* < 0.001, **** *p* < 0.0001.)

Wound healing activity of the developed propolis-loaded hydrogel was carried out with the burn wound model in comparison with standard 5% *w*/*w* povidone iodine ointment USP (Cipladine^®^). The developed formulation-treated group showed significant wound contraction compared with the untreated group as shown in Table 4 and Figure 6. The study results demonstrated the role of polyphenol, flavonoids, and oleic acid in down regulating COX 2, inducing collagen type II expression and thereby accelerating the wound healing process as suggested in the literature.

Propolis hydrogel G3 exhibited significant differences in wound contraction (*p* < 0.0001) as compared to blank hydrogel and 5% *w*/*w* povidone iodine ointment USP (Cipladine^®^).

At the end of the treatment period of 21 days, significant marked improvement in contraction of wound size was observed in the animals treated with propolis hydrogel (G3 93.58% ± 0.15% *p* < 0.0001) and 5% *w*/*w* povidone iodine ointment USP (Cipladine^®^) (95.39% ± 0.16%) as compared with blank hydrogel G2 58.38% ± 0.19% and disease control G1 52.37% ± 0.14%.The hydrogel formulation-treated group (G3) was found to be efficient in reducing the wound size and comparable to the standard group receiving 5% *w*/*w* povidone iodine ointment USP (Cipladine^®^).

**Figure 7:**
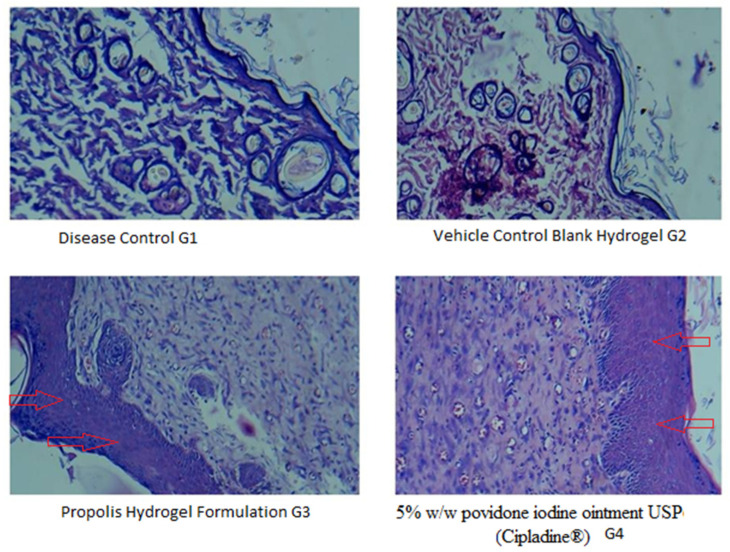
Photomicrograph in 40× lens showing histopathological changes of healed skin wounds on day 21 post wound (stained with hematoxyline and eosin). G1: diseased control; G2:vehicle control (blank hydrogel); G3: propolis hydrogel formulation; G4: treated with 5% *w*/*w* povidone iodine ointment USP (Cipladine^®^). Arrow shown indicates improved re-epithelialization.

Figure 7 shows the histopathological changes in the healing process of burned skin in various treatments. In all groups, the formation of collagen coir, re-epithelialization, and fat tissues was observed in the burn area. Wound healing has indicators which include collagen deposition, fibrosis, angiogenesis, and PMN infiltration. These parameters were revealed in varying levels in all four groups. Disease control (G1) showed the multifocal severe necrosis of the epidermis and dermis with infiltration of inflammatory cells and fibrous connective tissue. As compared to disease control and blank hydrogel, the propolis hydrogel and standard 5% *w*/*w* povidone iodine ointment USP (Cipladine^®^) showed a better response in terms of collagen deposition, development of epithelial lining, and neovascularization or angiogenesis, indicating an efficient wound healing process.

#### 2.8.2. Excision Wound Healing

##### Measurement of Wound Contraction

**Figure 8:**
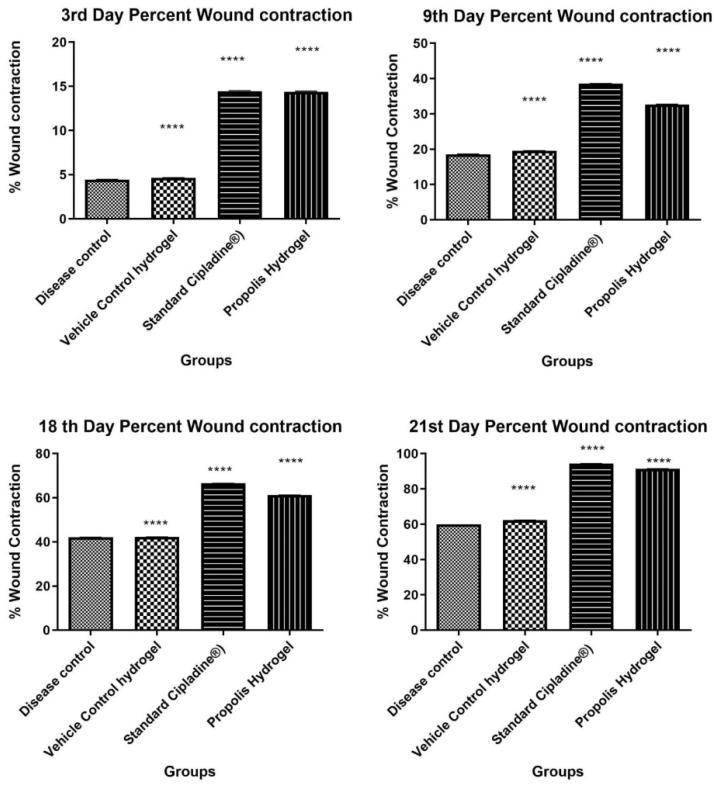
Percent wound contraction at time intervals of the 3rd, 9th, 18th, and 21st days (Data were analyzed by one-way ANOVA followed by Tukey–Kramer Multiple Comparison Test **** *p* < 0.0001).

Wound contraction of all the animals of the excision model was measured on the3rd, 9th, 18th, and 21st days after the creation of the wound. It was found that propolis hydrogel exhibits significant wound healing activity when compared against the standard and control groups (*** *p* < 0.0001), as shown in Table 5 and Figure 8.

##### Histopathological Studies

**Figure 9:**
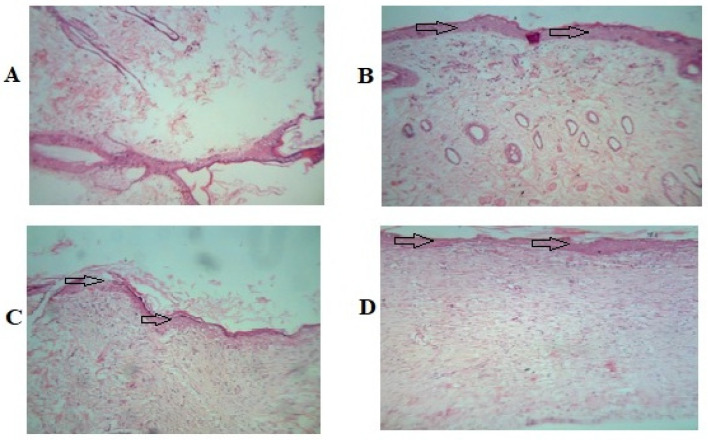
Photomicrographs in 40× lens of the histopathological section of wound tissue. (**A**) Group 1 (untreated group) animal wound tissue. (**B**) Group 2 (vehicle control) animal wound tissue. (**C**) Group 3 treated with 5% *w*/*w* povidone iodine ointment USP (Cipladine^®^). (**D**) Group 4 propolis hydrogel-treated animal wound tissue. Arrow shown indicates improved re-epithelialization.

The histopathological study of the wounded animal tissue is shown in the images below (Figure 9A–D).

The histopathological study reveals that Group 1 (A) has a lower presence of collagen fibers and blood vessels. In the image, some necrotic cells and inflammatory cells can be seen as well. In Group 2 (B), less collagen is visible compared to Group 4. The image of Group 3 (C) displays an increased number of fibroblastic cells, collagen fibers, and no necrotic changes. Images of Group 4 (D) show a significant amount of collagen deposition and a completely developed epithelial cell lining, which symbolizes wound healing.

#### 2.8.3. Incision Wound Healing

##### Measurement of Tensile Strength

**Figure 10:**
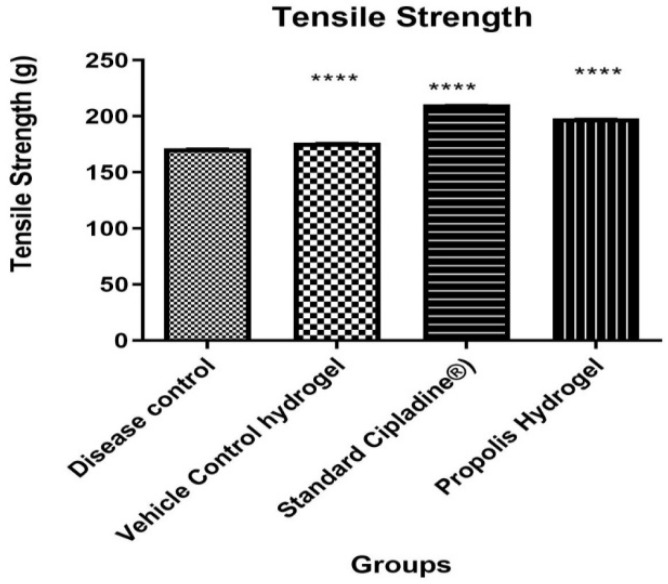
Effect of propolis hydrogel on tensile strength (Data were analyzed by one-way ANOVA followed by Tukey–Kramer Multiple Comparison Test **** *p* < 0.0001).

The tensile strength of the wound represents the effectiveness of wound healing. It is a measure of the completeness of wound healing and thus is an important parameter. From the results obtained, it was evident that the propolis hydrogel formulation showed significant tensile strength when compared against standard and control groups. Results of the tensile strength of the wound are represented in Table 6 and Figure 10.

### 2.9. Stability Studies

Accelerated stability studies of the formulations carried out at 40 ± 2 °C/75 ± 5% RH for a period of 6 months showed that the developed hydrogel does not exhibit any considerable changes in pH, viscosity, and drug content. Visual appearance showedno phase separation and it was found intact with no leakage during the period of six weeks at specified temperature ranges as shown in Table 7.

## 3. Conclusions

Indian propolis-loaded PVA hydrogel was developed, optimized, and evaluated for wound healing. A design of experiment approach was used to explore how critical formulation parameters affected dependent formulation variables. Propolis samples were extracted utilizing literature-based extraction methods to increase polyphenols and flavonoids for wound healing. A validated RP HPLC method standardized the extract for specified constituents. The extract has 34.82 + 0.072 mg equivalent of gallic acid/g and 23.61 + 0.045 mg equivalent of quercetin/g, which may act synergistically. The extract was also free of 113 pesticides, proving its safety for drug delivery. Formulation development was conducted using a 3^2^ factorial design to determine how independent variables (critical material attributes and process parameters) impact dependent variables (in vitro drug release and viscosity). Design Expert^®^ Version 10.0 was used for multiple regression analysis for batch optimization. The formulation batch (F2) was optimized utilizing the software’s highest desirability of 1.000 at the desirable in vitro drug release and viscosity. FTIR and DSC analysis showed uniform distribution of propolis in hydrogel composition. Acute toxicity studies and burn, excision, and incision wound healing models show safety and substantial wound healing (*p* < 0.0001). Overall research results indicate promising finished product specifications of a developed formulation that could be studied for therapeutic effect in clinical trials.

## 4. Material and Methods

### 4.1. Chemicals

Crude propolis was purchased from Bharatpur, Rajasthan, India. Polyvinyl alcohol LR was purchased from Research Lab Fine Chem Industries (Mumbai, India). Dialysis bags with a molecular weight cut off of 12,000 Dalton were purchased from Sigma-Aldrich Chemical Private Ltd. (Bangalore, India). All other chemical reagents used were of analytical grade.

### 4.2. Extraction and Standardization of Propolis Extract

The propolis sample was first treated with hexane to remove wax and other debris. The sample was then extracted with ethanol to obtain an extract high in polyphenols and flavonoids and desired groups of chemical constituents and standardized with the developed and validated RP HPLC method as described in our previously published work [24].

The extract was also standardized with the developed and validated RP-HPLC method with respect to specific marker compounds caffeic acid, quercetin, apigenin, and caffeic acid phenethyl ester [24]. The extract was also evaluated for its polyphenol, flavonoid, and pesticide contents.

### 4.3. FTIR Study

FTIR studies were carried out with the KBR dispersion technique for identification of specific functional groups from propolis extract and formulation. The spectrum was recorded and the spectral analysis was conducted (Shimadzu Model-8400S) [25].

### 4.4. DSC

The differential scanning calorimetry (DSC) thermograms of propolis extract and formulation were conducted on a DSC 821e (Mettler-Toledo, Greifensee, Switzerland). Samples were (5 mg) heated in a hermetically sealed aluminum pan with a heating rate of 10 °C/min under a nitrogen atmosphere (flow rate 50 mL/min) [26].

### 4.5. Formulation Development of Hydrogel and Optimization by DOE

A 10% *w*/*v* PVA aqueous solution was prepared (60–90 °C) under mechanical stirring for 6 h, and this solution was kept under stirring until it reached room temperature. For the PVA–propolis hydrogel formulation, when the PVA solution reached room temperature, 4 gm of ethanolic extract of propolis was added under mechanical stirring. Further formulation was then freeze–thawed for 24 h at −18 °C followed by 5–7 cycles of 30~60 min at room temperature and 1 h at −18 °C [19,20,27].

Then, 3^2^ factorial designs were used to carry out experimental runs to understand the impact of variables on responses. The concentration of PVA and stirring speed were selected as independent variables, whereas drug release and viscosity were selected as dependent variables. The resulting data were fitted into design expert software (DOE version 10) and statistically analyzed using analysis of variance (ANOVA). The data were also subjected to 3D response surface methodology to determine the influence of material and process attributes on responses. The designs of the experimental runs are shown in Table 1.

### 4.6. Characterization of Hydrogel

#### 4.6.1. Physical Appearance, pH, and Viscosity

The prepared gel formulation was observed for a period of 6 months (0, 1, 2, 3, 6 months) for physical appearances such as homogeneity, color, consistency, grittiness and separation, etc. The pH of the gel formulation was determined by using a digital pH meter (Systronics pH meter, Type 335) [28].Viscosity was determined with a Brook field RVDV-II + Pro viscometer with a small volume adaptor spindle (S96) and T-bar spindle. All experimentation was carried out in triplicate and average values were calculated [29,30].

#### 4.6.2. In Vitro Drug Release Study

The release of Indian propolis from the hydrogel formulation was assessed by the dialysis bag diffusion technique with phosphate buffer at pH 7.4 as a release medium at 37 ± 0.5 °C with the continuous stirring process at 100 rpm. A propolis hydrogel sample equivalent to 80 mg was filled in the dialysis bag membrane (molecular weight cut off of 12,000 Da) and the bag was immersed in release medium. Then, 2 mL of the aliquots were withdrawn at different time points: 0, 15 min, 30 min, 1 h, 2 h, 3 h, 4 h, and 6 h. Thereafter, aliquots were filtered through Whatman no.1 filter paper, and the obtained solution was diluted and the propolis concentration was estimated. The pure Indian propolis release pattern was analyzed in a similar manner, taking Indian propolis solution (1 mg/mL in 50% *w*/*w* mixture of PEG 400 and water) as a control [31].

#### 4.6.3. Acute Dermal Toxicity

The study was conducted according to the OECD guideline 434 (11). The extract at a dose of 2000 mg/kg of body weight was administered dermally as a single dose to Albino Wistar rats (male and females; 180–200 gm body weight) and allowed to keep in contact with the skin for 24 h. Animals were carefully observed for the first 30 min followed by 24 h periodically, and monitoring continued until14 days. Animals were observed for any rashes on skin, changes in skin, eye changes, respiratory changes, nervous system changes, behavioral changes, locomotor activity, convulsions, tremors, comas, etc. Percent body weight changes in the animals were recorded [32].

#### 4.6.4. Wound Healing Activity

##### Burn Wound Model

Male Wistar rats weighing 150–250ginthe age range of three to four months were used in the study. Animals were procured from the National Toxicological Centre (NTC), Pune, India, and were housed in an animal house at room temperature, being maintained at 37 ± 5 °C. Animals were fed on a standard diet. Animals were given free access to food and water. All the experiments were performed in accordance with guidelines laid by the Committee for Purpose of Control and Supervision on Experiments on Animals (CPCSEA) and approved by the Institutional Animal Ethics committee as per protocol number DYPIPSR/IAEC/14-15/P-34.

The animals were randomly divided into 4 groups (n = 6). The animals were anesthetized using ketamine hydrochloride (50 mg/kg, i.p., body weight) and xylazine (5 mg/kg, i.p., body weight). Group 1 (disease control) animals were animals that were wounded but did not receive treatment, Group2 (vehicle control group) was treated with blank PVA hydrogel without drugs, Group 3 was treated with the propolis hydrogel formulation, and Group 4 animals received treatment of standard 5% *w*/*w* povidone iodine ointment USP(Cipladine^®^). Groups 1–4 were treated once daily for a period of 21 days. The animals were kept in separate cages and were provided a standard diet and water adlibitum throughout the study. The rate of wound contraction was noted. At the end of the study, the animals were sacrificed and wound tissue mass was obtained from the wound area by sharp dissection [21,27].

Percent wound contraction was measured with a digital vernier caliper by calculation with the formula mentioned below and taking the initial size of the wound as 100%.

Wound size reduction (%) = [A_0_ − At]/A_0_ × 100

where A_0_: initial size of wound contraction, At: final size of wound contraction [20,21].

Histopathological Studies were carried out to confirm healing improvement. The wound tissue specimens were processed in 5% formalin solution embedded in paraffin. A section of 3–5 cm of skin epidermis tissue was stained with hematoxylin and eosin (H & E) for routine histological processing. The prepared slides were examined qualitatively under a light microscope, for examining hair follicles, inflammation, blood vessels, fibroblast, necrosis, bacterial colonies, neutrophils, edema, and collagen [21,27].

##### Excision and Incision Wound Model

The excision wound model was used to measure the wound contraction and for histopathological study, while the incision wound model was used to find out the tensile strength of the cured tissue of rats.

The animals were grouped as follows: Group 1, disease control; Group 2, vehicle control; Group 3, standard control, treated with 5% *w*/*w* povidone iodine ointment USP (Cipladine^®^); Group 4, treated with propolis hydrogel. Each group contained a total of 6 animals.

Excision Wound Model was studied with four groups containing 3 animals each were made. The animals were anesthetized using ketamine hydrochloride (50 mg/kg, i.p., body weight) and xylazine (5 mg/kg, i.p., body weight). The dorsal portion of the rats was shaved using depilatory cream. The area to be excised was marked and a full-thickness excision of 1 cm diameter was made [32,33]. The wounds were left undressed. Vehicle control (blank PVA hydrogel), standard 5% *w*/*w* povidone iodine ointment USP (Cipladine^®^), and propolis hydrogel were applied once daily until the wound had completely healed. Wound contraction was measured on the 3rd, 9th, 18th, and 21st days after the formation of the wound. After the study had been completed, i.e., on the 21st day after the wound creation, the rats were anesthetized. Tissues of rat wounds were excised and stored in 10% formalin solution for histopathological study [33,34].

Incision Wound Model was studied with 4 groups of animals containing 3 animals each were made. All the animals were anesthetized (using the same anesthetic agent used above) and depilated using depilatory cream. An incision of 3 cm length was made according to the method described by Mukherjee et al., 2000. The parted skin was kept together by stitching using a curved needle (No. 9) and black surgical thread (No.000). Vehicle control (blank PVA hydrogel), standard 5% *w*/*w* povidone iodine ointment USP (Cipladine^®^), and propolis hydrogel were applied to the wound area once daily for 9 continuous days. On the 10th day, when the wound had healed, the animals were anesthetized again and the sutures were removed. The tensile strength of the healed wound was measured using a tensiometer [35].

The tensile strength of the wound is an indication of the effectiveness of wound healing. Tensile strength, i.e., the force required to open the healing wound, is used as an important evaluation parameter. On the 10th day, the rats of the incision model were anesthetized, the sutures were removed, and healed tissue was excised from all animals. The tensile strength of this excised tissue was measured with the help of a tensiometer [35].

#### 4.6.5. Statistical Analysis

The results obtained from the excision and incision models were expressed as mean ± standard error of the mean (SEM) and were compared with the vehicle control, disease control, and standard control groups. The statistical significance was analyzed by one-way ANOVA and the Tukey–Kramer Multiple Comparison Test, using statistical software Graph Pad Prism, version 5.

#### 4.6.6. Stability Testing of Hydrogel Formulation

Accelerated stability studies of the propolis hydrogel formulation were carried out as per ICH guidelines at conditions of 40 °C ± 2 °C/75 ± 5% RH for a period of 6 months. The stability samples (n = 3) were analyzed for drug content, pH, and viscosity at 0, 30, 60, 90, and 180 days [36].

## Figures and Tables

**Table 1:** Experimental runs by 3^2^ factorial designs.

Batch Codes	Polymer Concentration X1	Stirring Speed X2	Polymer Concentration (%) X1	Stirring Speed (rpm) X2
	Code	Code	Actual	Actual
F1	+1	+1	10	800
F2	+1	0	10	700
F3	+1	−1	10	600
F4	0	+1	09	800
F5	0	0	09	700
F6	0	−1	09	600
F7	−1	+1	08	800
F8	−1	0	08	700
F9	−1	−1	08	600

**Table 2:** Effects of formulation variables on in vitro drug release and viscosity.

Batch No	Polymer Concentration X1	Stirring Speed X2	Response 1	Response 2
			% Cumulative Drug Release (4 h)	Viscosity cps
Unit	%	rpm	%	cps
F1	+1	+1	88.16	32,500
F2	+1	0	87.89	33,600
F3	+1	−1	86.16	34,200
F4	0	+1	92.11	29,900
F5	0	0	96.89	30,100
F6	0	−1	94.26	30,900
F7	−1	+1	93.25	24,200
F8	−1	0	97.90	25,600
F9	−1	−1	95.36	26,300

**Table 3:** Visual examination of prepared gel formulation.

Sr.N	Colour	Homogeneity	Consistency	Grittiness	Phase Separation
F2	Brownish	Homogenous	Consistent	Non-greasy	No phase separation

**Table 4:** Percent wound contraction at time intervals of the 3rd, 9th, 18th, and 21st days.

Group Number	Name of Group	Day 3	Day 9	Day 18	Day 21
1.	Disease control	2.45 ± 0.11	17.21 ± 0.08	41.26 ± 0.11	52.37 ± 0.14
2.	Vehicle control hydrogel	3.32 ± 0.15	20.29 ± 0.10	46.32 ± 0.12	58.38 ± 0.19
3.	Propolis hydrogel	12.39 ± 0.16 a ****, b ****, c ****	31.51 ± 0.12 a ****, b ****, c ****	73.50 ± 0.27 a ****, b ****, c ****	93.58 ± 0.15 a ****, b ****, c ****
4.	5% *w*/*w* povidone iodine ointment USP (Cipladine^®^)	13.44 ± 0.13	33.46 ± 0.06	76.33 ± 0.26	95.39 ± 0.16

All values are represented as mean ± SEM, n = 6 animals in each group. Data were analyzed by one-way ANOVA followed by Tukey–Kramer Multiple Comparison Test. a: significant difference compared to Group 1. b: significant difference compared to Group 2. c: significance compared to Group 4. **** *p* < 0.0001.

**Table 5:** Percent wound contraction at time intervals of the 3rd, 9th, 18th, and 21st days.

Groups	% Wound Contraction			
	3rd Day	9th Day	18th Day	21st Day
Group 1: Disease control	4.41 ± 0.13	18.61 ± 0.94	42.09 ± 0.10	59.95 ± 0.07
Group 2: Vehicle control	4.64 ± 0.07	19.57 ± 0.07	42.29 ± 0.67	62.30 ± 0.40
Group 3: 5% *w*/*w* povidone iodine ointment USP (Cipladine^®^)	14.45 ± 0.16	38.63 ± 0.07	66.57 ± 0.12	94.38 ± 0.21
Group 4: Propolis hydrogel	14.39 ± 0.13 a ****, b ****, c ****	32.69 ± 0.13 a ****, b ****, c ****	61.26 ± 0.03 a ****, b ****, c ****	91.45 ± 0.29 a ****, b ****, c ****

All values are represented as mean ± SEM, n = 6 animals in each group. Data were analyzed by one-way ANOVA followed by Tukey–Kramer Multiple Comparison Test. a: significant difference compared to Group 1. b: significant difference compared to Group 2. c: significance compared to Group 3. **** *p* < 0.0001.

**Table 6:** Effect of propolis hydrogel on tensile strength.

Groups	Tensile Strength in g
Group1: Disease control	171.40 ± 0.17
Group 2: Vehicle control	176.49 ± 0.21
Group 3: 5% *w*/*w* povidone iodine ointment USP (Cipladine^®^)	210.46 ± 0.19
Group 4: Propolis hydrogel	198.18 ± 0.22 a ****, b ****, c ****

All values are represented as ±SEM, n = 6 animals in each group. Data were analyzed by using one-way ANOVA, followed by Tukey–Kramer Multiple Comparison Test. a: significant difference compared to Group 1. b: significant difference compared to Group 2. c: significant difference compared to Group 3. **** *p* < 0.0001.

**Table 7:** Accelerated stability study of hydrogel formulation.

	Propolis Hydrogel Formulation				
Evaluation Parameters	0 Days	30 Days	60 Days	90 Days	180 Days
Physical Appearance	Dark brownish, homogeneous, non-greasy				
Phase separation	Nil	Nil	Nil	Nil	Nil
pH	5.46 ± 0.246	5.44 ± 0.142	5.44 ± 0.554	5.41 ± 0.311	5.41 ± 0.110
Drug content	98.96%	98.60%	98.52%	98.51%	98.12%
Viscosity	31,500 cps	31,450 cps	31,400 cps	31,440 cps	31,400 cps

## Data Availability

Not applicable.

## References

[1] Kapare H.S., Sathiyanarayanan L. (2020). Nutritional and Therapeutic potential of Propolis: A Review. Res. J. Pharm. Tech..

[2] Kapare H.S., Sathiyanarayanan L., Mahadik K.R., Arulmozhi S. (2017). Indian Propolis Loaded Folic Acid Conjugated PLGA Nanoparticles: Formulation Development, Characterization, In Vitro and In Vivo Anticancer Study. J. Pharm. Drug Deliv. Res..

[3] Wagh V.D., Borkar R.D. (2012). Indian propolis: A potential natural antimicrobial and antifungal agent. Int. J. Pharm. Pharm. Sci..

[4] Krol W., Czuba Z., Scheller S., Gabrys J., Grabiec S., Shani J. (1990). Anti-oxidant property of ethanolic extract of propolis (EEP) as evaluated by inhibiting the chemiluminescence oxidation of luminol. Biochem. Int..

[5] Li H., Kapur A., Yang J.X., Srivastava S., McLeod D.G., Paredes-Guzman J.F. (2007). Antiproliferation of human prostate cancer cells by ethanolic extracts of Brazilian propolis and its biological origin. Int. J. Oncol..

[6] Kumar M.R., Bose V.S.C., Sathyabama S., Priyadarshini V.B. (2011). Antimicrobial and DPPH free radical-scavenging activities of the ethanol extract of propolis collected from India. J. Ecobiotechnol..

[7] Cai R., Gimenez-Camino N., Xiao M., Bi S., Kyle A. (2023). Technological advances in three-dimensional skin tissue engineering. Rev. Adv. Mater. Sci..

[8] Kapare H.S., Metkar S.R., Wakalkar S.V. (2020). Natural products in wound healing: Nano-technology based approaches. Indian Drugs.

[9] Yazarlu O., Iranshahi M., Kashani H.R.K., Reshadat S., Habtemariam S., Iranshahy M., Hasanpour M. (2021). Perspective on the application of medicinal plants and natural products in wound healing: A mechanistic review. Pharm. Res..

[10] Sen C.K. (2019). Human Wounds and Its Burden: An Updated Compendium of Estimates. Adv. Wound Care.

[11] Stanicka K., Dobrucka R., Woźniak M., Sip A., Majka J., Kozak W., Ratajczak I. (2021). The Effect of Chitosan Type on Biological and Physicochemical Properties of Films with Propolis Extract. Polymers.

[12] da Rosa C., Bueno I.L., Quaresma A.C.M., Longato G.B. (2022). Healing Potential of Propolis in Skin Wounds Evidenced by Clinical Studies. Pharmaceuticals.

[13] Oryan A., Alemzadeh E., Moshiri A. (2018). Potential role of propolis in wound healing: Biological properties and therapeutic activities. Biomed. Pharm..

[14] Stojko M., Wolny D., Włodarczyk J. (2021). Nonwoven Releasing Propolis as a Potential New Wound Healing Method-A Review. Molecules.

[15] Yang J., Pi A., Yan L., Li J., Nan S., Zhang J., Hao Y. (2022). Research Progress on Therapeutic Effect and Mechanism of Propolis on Wound Healing. Evid. Based Complement Altern. Med..

[16] Martinotti S., Ranzato E. (2015). Propolis: A new frontier for wound healing?. Burns Trauma.

[17] Wojciech S., Aleksandra G., Aleksandra K., Filip C., Sławomir B., Hanna Maria B., Katarzyna W., Ewa K., Maciej J. (2023). Design of vitamin-loaded emulsions in agar hydrogel matrix dispersed with plant surfactants. Food Biosci..

[18] Pardeshi S., Damiri F., Zehravi M., Joshi R., Kapare H., Prajapati M.K., Munot N., Berrada M., Giram P.S., Rojekar S. (2022). Functional Thermoresponsive Hydrogel Molecule to Material Design for Biomedical Applications. Polymers.

[19] Peppas N.A., Merrill E.W. (1976). Differential scanning calorimetry of crystallized PVA hydrogels. J. Appl. Polym. Sci..

[20] Hickey A.S., Peppas N.A. (1995). Mesh size and diffusive characteristics of semicrystalline poly (vinyl alcohol) membranes prepared by freezing/thawing techniques. J. Membr. Sci..

[21] Khan A.W., Kotta S., Ansari S.H., Sharma R.K., Kumar A., Ali J. (2013). Formulation development, optimization and evaluation of aloe vera gel for wound healing. Pharm. Mag..

[22] Wang M., Bai J., Shao K., Tang W., Zhao X., Lin D., Huang S., Chen C., Ding Z., Ye J. (2021). Poly(vinyl alcohol) Hydrogels: The Old and New Functional Materials. Int. J. Polym. Sci..

[23] Afkhamizadeh M., Aboutorabi R., Ravari H., Fathi Najafi M., Ataei Azimi S., Javadian Langaroodi A., Yaghoubi M.A., Sahebkar A. (2018). Topical propolis improves wound healing in patients with diabetic foot ulcer: A randomized controlled trial. Nat. Prod. Res..

[24] Kapare H.S., Lohidasan S., Arulmozhi S., Mahadik K.R. (2019). Standardization, chemical profiling, *in vitro* cytotoxic effects, *in vivo* anti-carcinogenic potential and biosafety profile of Indian propolis. J. Ayurveda Integr. Med..

[25] Kapare H.S., Sathiyanarayanan L., Arulmozhi S., Mahadik K.R. (2020). Caffeic Acid Phenethyl Ester Loaded Poly (ε –caprolactone) Nanoparticles for Improved Anticancer Efficacy: Formulation Development, Characterization and *in Vitro* Cytotoxicity Study. Nanomed. Res. J..

[26] Kapare H.S., Lohidasan S., Sinnathambi A., Mahadik K. (2021). Formulation Development of Folic Acid Conjugated PLGA Nanoparticles for Improved Cytotoxicity of Caffeic Acid Phenethyl Ester. Pharm. Nanotechnol..

[27] Oliveira R.N., McGuinness G.B., Rouze R., Quilty B., Cahill P., Soares G.D., Thiré R.M. (2015). PVA hydrogels loaded with a Brazilian propolis for burn wound healing applications. J. Appl. Polym. Sci..

[28] Peppas N.A., Merrill E.W. (1976). PVA hydrogels: Reinforcement of radiation-crosslinked networks by crystallization. J. Polym. Sci. Polym. Chem. Ed..

[29] Wang F., Gao Y., Li H., Zhou L., Shi H., Feng S., Chen J., Mei Z. (2022). Effect of natural-based biological hydrogels combined with growth factors on skin wound healing. Nanotechnol. Rev..

[30] Stauffer S.R., Peppas N.A. (1992). Poly (vinyl alcohol) hydrogels prepared by freezing thawing cyclic processing. Polymer.

[31] Hua S. (2014). Comparison of *in vitro* dialysis release methods of loperamide-encapsulated liposomal gel for topical drug delivery. Int. J. Nanomed..

[32] Organisation for Economic Co-operation and Development (OECD) (2017). Test No. 402: Acute Dermal Toxicity, OECD Guidelines for the testing of Chemicals, Section 4.

[33] Nagar H.K., Srivastava A.K., Srivastava R., Kurmi M.L., Chandel H.S., Ranawat M.S. (2016). Pharmacological investigation of the wound healing activity of *Cestrum nocturnum* (L.) ointment in Wistar albino rats. J. Pharm..

[34] Hemalatha S., Subramanian N., Ravich V., Chinnaswamy K. (2001). Wound healing activity of *Indigoferaenneaphylla* Linn. Indian J. Pharm. Sci..

[35] Mukherjee P.K., Verpoorte R., Suresh B. (2000). Evaluation of in-vivo wound healing activity of Hypericumpatulum (Family: Hypericaceae) leaf extract on different wound model in rats. J. Ethnopharmacol..

[36] Qindeel M., Ahmed N., Sabir F., Khan S., Ur-Rehman A. (2019). Development of novel pH-sensitive nanoparticles loaded hydrogel for transdermal drug delivery. Drug Dev. Ind. Pharm..

